# The TBAg/PHA ratio in T-SPOT.TB assay has high prospective value in the diagnosis of active tuberculosis: a multicenter study in China

**DOI:** 10.1186/s12931-021-01753-5

**Published:** 2021-06-01

**Authors:** Yidian Liu, Lan Yao, Feng Wang, Ziyong Sun, Yaoju Tan, Wei Sha

**Affiliations:** 1 Department of Tuberculosis, Shanghai Pulmonary Hospital, Tongji University School of Medicine, No. 507, Zhengmin Road, Shanghai, 200433 China; 2 Shanghai Clinical Research Center for Infectious Disease (Tuberculosis), Shanghai, 200433 China; 3 Department of Laboratory Medicine, Tongji Hospital, Tongji Medical College, Huazhong University of Science and Technology, Wuhan, China; 4 Department of Clinical Laboratory, Guangzhou Chest Hospital, Guangzhou, China

**Keywords:** TBAg/PHA ratio, T-SPOT, Active tuberculosis, Non-tuberculosis, Diagnosis

## Abstract

**Background:**

The positive rate of pathogenic examination about tuberculosis is low. It is still difficult to achieve early diagnosis for some TB patients. The value of Interferon-gamma release assays (IGRA) in the diagnosis of active tuberculosis remains controversial. The purpose of this multicenter prospective study was to verify and validate the role of TBAg/PHA ratio (TB-specific antigen to phytohaemagglutinin) of T-SPOT.TB assay in diagnosing ATB.

**Methods:**

We prospectively enrolled 2390 suspected pulmonary tuberculosis patients with positive T-SPOT assay results from three tertiary hospitals.

**Results:**

A total of 1549 ATB (active tuberculosis) patients (including 1091 confirmed and 458 probable ATB) and 724 non-tuberculosis (non-TB) patients with positive T-SPOT results were included. The results of this study showed that ESAT-6 and CFP-10 in the T-SPOT.TB assay were significantly higher in the ATB group compared with the non-TB group, while PHA was lower in the ATB group. Results of ESAT-6, CFP-10 and PHA show a certain diagnostic performance, but moderate sensitivity and specificity. The TBAg/PHA ratio, a further calculation of ESAT-6, CFP-10 and PHA in T-SPOT.TB assay showed improved performance in the diagnosis of active Tuberculosis. If using the threshold value of 0.2004, the specificity and sensitivity of TBAg/PHA ratio in distinguishing ATB from non-TB were 92.3% and 74.4%, PPV was 95.4, PLR was 9.6.

**Conclusion:**

By recalculating the results of T-SPOT.TB Assay, the TBAg/PHA ratio shows high prospect value in the diagnosis of active tuberculosis in high prediction areas.

## Introduction

Tuberculosis (TB) is still one of the main causes of mortality worldwide. It is estimated that one-third of the world's population is infected with Mycobacterium tuberculosis (Mtb) [1]. Globally in 2018, an estimated ten million people fell ill with TB [2]. Early diagnosis of pulmonary TB is crucial for preventing the spread of Mtb infection. However, it is still difficult to achieve early diagnosis and timely treatment for some TB patients. Diagnostic assessment of suspected tuberculosis can also be lengthy, costly, and burdensome for patients and health-care systems [3].

Conventional TB diagnosis continues to rely on smear microscopy, mycobacterium culture and chest radiography. These tests have known limitations [4–6]. The polymerase chain reaction (PCR)-based GeneXpert MTB/RIF sputum assay improves the speed and specificity of TB diagnosis, but still has limited sensitivity under low bacterial loads [7, 8]. Therefore, GeneXpert MTB/RIF assay requires clinical specimens from anatomical disease sites, often requiring resource-intensive invasive procedures [9].

T-cell interferon-γ release assays (IGRAs) are based on interferon-γ (IFN-γ) secretion by lymphocytes exposed to M. tuberculosis-specific antigens (TBAg). IGRAs has been proven useful in the detection of Mtb infection [10–12]. Because of the good performance, IGRAs was established for the immunodiagnosis of latent tuberculosis infection (LTBI) in many countries, and recommended by WHO [13]. The analysis showed that the sensitivity of IGRAs was not high enough to use as a rule out test for TB [14]. But a number of different studies on IGRAs in the diagnosis of active tuberculosis (ATB) have been published in recent years, suggesting that the role of IGRA in the diagnosis of ATB is still worth further evaluation [15, 16], some prospective studies are needed.

T-SPOT.TB (T-SPOT) assay, as one of two commercially available IGRAs, is widely used in clinical practice. The degree to which the magnitude of the T-SPOT.TB results were found to be determined largely by the host immune response [17], and speculated to be associated with mycobacterial antigen load (which reflects bacterial load) [18, 19]. Some studies report that T-SPOT has certain diagnostic value for active tuberculosis, and has higher diagnostic accuracy of active TB [14, 16], but the specificity and sensitivity have not yet reached practical application. We found that phytohemagglutinin (PHA) results in T-SPOT assay can reflect the immune status of the patient [20]. Our group has previously shown that calculation of the ratio of MTB-specific antigen (TBAg) to phytohaemagglutinin (PHA) (TBAg/ PHA ratio) in T-SPOT assay has the potential to distinguish certain types of ATB from LTBI [21, 22]. That was a retrospective and case–control study.

Therefore, we conducted a prospective study to validate the diagnostic value of TBAg/PHA ratio for active tuberculosis. Meanwhile, we also provide important up-to-date information for further using T-SPOT to distinguish active tuberculosis from LTBI.

## Materials and methods

### Study design and participants

This study was a prospective cohort study. It validates the diagnostic value of TBAg/PHA ratio for active tuberculosis based on the patient’s final diagnosis. It was also a real-world clinical practice and the enrollment of the subjects was continuous from the beginning of the study. This study was conducted at three tertiary hospitals including Shanghai Pulmonary Hospital, Tongji Hospital and Guangzhou Chest Hospital in China. The hospitals are located in the east, center, and south of China. Between October 2016 and October 2017, patients who were suspected with pulmonary TB and had undergone the T-SPOT.TB test, and older than 16 years were enrolled in the study at three hospitals. Patients with suspected pulmonary TB mean he/she have TB-related symptoms (fever, fatigue, chest pain, cough, night sweats, weight loss, productive sputum) and abnormal pulmonary radiographic evidence consistent with active pulmonary TB (lung infiltrations, fibronodular lesions and cavitary lesion).

Participants were excluded from this study if they had (1) negative T-SPOT.TB result; (2) indeterminate T-SPOT results; (3) patients refused to do the requested tests.

### Study groups

All participants have clinical and radiological features suggestive of tuberculosis, and the diagnose was categorised into the following groups: (1) ATB: including confirmed TB and probably TB; (2) Confirmed ATB: Mycobacterium tuberculosis was confirmed by mycobacterium culture or GeneXpert MTB/RIF assay of clinical specimens; (3) Probable ATB: the clinical and radiological findings were highly suggestive of TB, but both the results of mycobacterium culture and GeneXpert MTB/RIF assay were negative, and were appropriate response to anti-TB treatment after a follow-up of at least 2 months according to the decision of the clinician; (4) non-tuberculosis (non-TB): refers to patients with radiological abnormalities but GeneXpert MTB/RIF negative and mycobacterium culture negative, and patients had final diagnosis as other diseases (cancer, pneumonia, bronchiectasis etc.) after clinical examination or necessary treatment, and were no clinical basis for active TB. Non-TB in this study were individual who had other diseases and also LTBI. The final diagnosis and diagnosis category were decided by the experts of respiratory and infectious diseases. Participants without a final diagnosis meant that the disease still could not be confirmed until the follow-up of 6 months, but active tuberculosis was excluded.

### Clinical and laboratory procedures

Participants were investigated in routine practice under the direction of the infectious disease or respiratory medicine attending physician. Patient’s data were collected of the demographics and medical history of the participant and subsequent investigations performed during routine diagnostic work-up. Bronchial washing fluid was collected from the lung segment that showed abnormal lesions on CT or chest X-ray. Sputum samples and other clinical specimens were collected for performing mycobacterium culture, GeneXpert MTB/RIF assay, AFS (Ziehl–Neelsen staining), pathological examination, general bacteriological culture, fungal culture, etc.

The patients with negative Mtb results will undergo further examination and necessary treatment based on the consideration of clinicians. In some patients, bronchoscopy biopsy or CT-guided biopsy were performed to assist the diagnosis if necessary. If other diseases were excluded, the clinician still considers active TB to be highly possibility, anti-TB treatment will be started after full communication and consent with the patient. Participants were followed up for 2 months or 6 months to collect data when necessary. If antituberculosis treatment was initiated, their response was recorded and followed up.

Peripheral blood T-SPOT assay was performed according to the manufacturer's instructions (Oxford Immunotec, Oxford, England) in three hospitals. T-SPOT.TB test was performed according to the manufacturers instructions. The results were double-checked if necessary, and corrected by manual counting. The laboratory technicians were blinded to the subject identifiers. Calculation of TBAg/PHA ratio: the ratios of (1) ESAT-6 sfc to PHA sfc, and (2) CFP-10 sfc to PHA sfc were calculated. The higher of these two values was defined as the TBAg/PHA ratio for one patient.

### Statistical analysis

Statistical analysis was performed using the statistical software GraphPad Prism (version 5.01; GraphPad, La Jolla, CA, USA.) and SPSS (version 21.0; IBM Corp, Chicago, IL, USA). Differences between groups were analysed using the Mann–Whitney U-test. Receiver operating characteristic (ROC) analysis was performed to determine the best cut off value for TBAg/PHA ratio, ESAT-6 sfc, CFP-10 sfc, and PHA sfc in distinguishing ATB from non-TB. Area under the curve (AUC) and cutoff values with the highest sum of sensitivity and specificity were identified. Sensitivity, specificity, positive and negative predictive values (PPV, NPV), and positive and negative likelihood ratios (PLR, NLR) for each threshold value were calculated. The sensitivity and specificity were calculated, along with 95% confidence interval (CI), using the Wilson score method. Spearman’s rank correlation test for non-parametric data was employed to analyze the relationship between two factors. Statistical significance was determined as p < 0.05.

## Results

### Study population and clinical characteristics

**Fig. 1:**
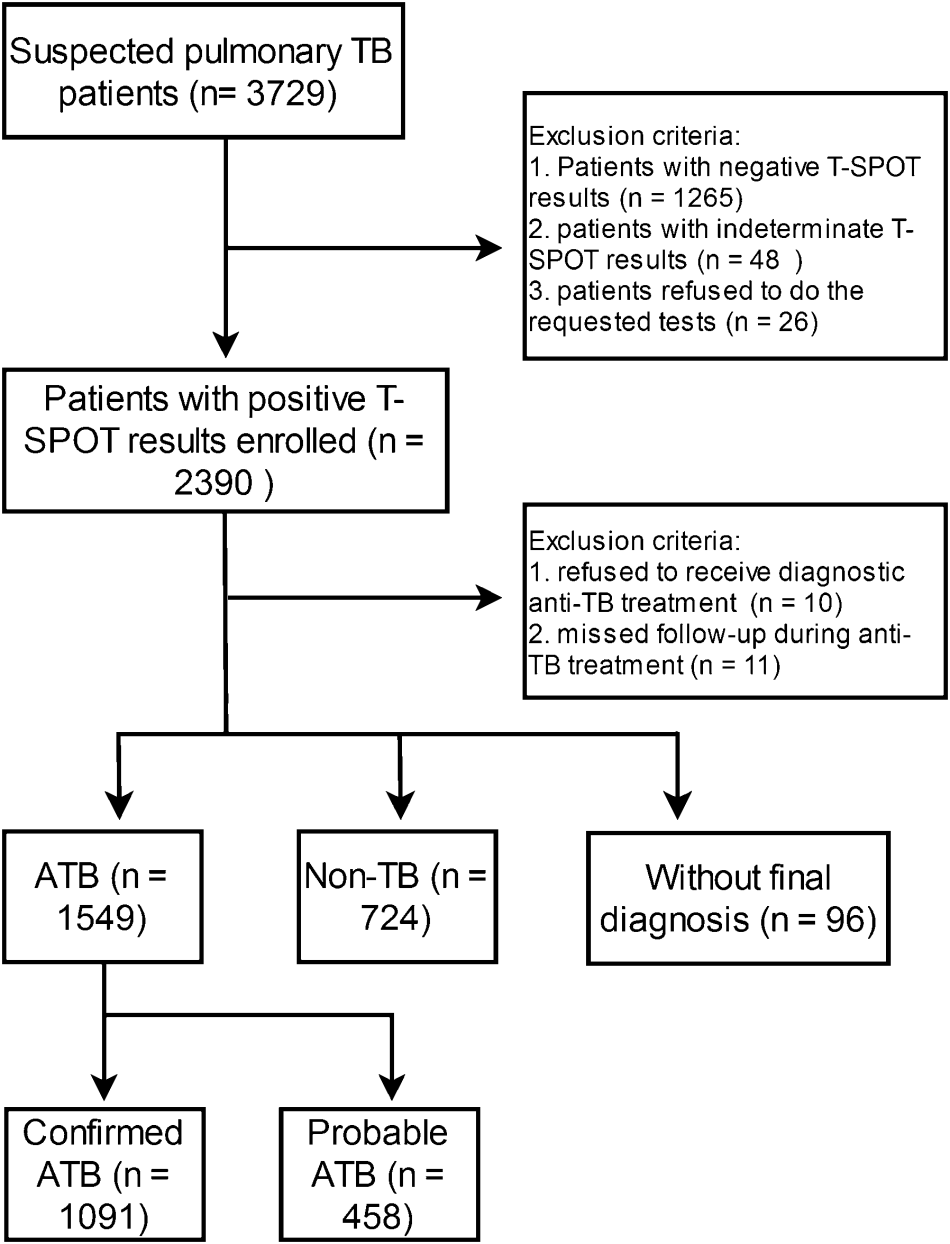
Flowchart of study data

A total of 3729 subjects with suspected diagnosis of pulmonary TB were recruited from three tertiary hospitals. Finally, there were 2390 (64.09%) patients with positive T-SPOT results were enrolled in the study. The patients (n = 10) were refused to receive diagnostic anti-TB treatment, and the patients (n = 11) were lost follow-up during anti-TB treatment. Among these patients, 1549 (64.8%) patients were finally diagnosed with ATB, including 1091(70.4%) confirmed TB cases, and 458 (29.6%) probable ATB (Fig. 1). There were 724 (30.3%) patients were finally diagnosed with non-TB. Among the 724 non-TB cases, including 39.2% (284/724) bacterial pneumonia, 16.3% (118/724) bronchiectasis, 12.3% (89/724) lung cancer, 11.8% (86/724) nontuberculous mycobacteria infection, 7.3% (53/724) fungal infection, 4.6% (33/724) virus infection and 1.9% (14/724) parasitic infection; and 6.4% (47/724) other diseases were included. 96 (0.04%) patients had no final diagnosis (Fig. 1). The demographic and clinical characteristics of the participants are shown in Table 1.

**Table 1:** Demographic and clinical characteristics of ATB and non-TB patients

Characteristic	ATB (n = 1549)	Non-TB (n = 724)	P value
Mean age (mean ± SD), years	45.56 ± 16.72	47.31 ± 17.25	
Male sex	1052 (67.91)	456 (62.98)	0.053
History of TB	278 (17.95)	23 (3.18)	< 0.01
Immunosuppressive conditions	382 (24.66)	125 (17.27)	< 0.01
Human immunodeficiency virus infection	8 (0.52)	1 (0.14)	0.287
Hematologic malignancy	21 (1.36)	5 (0.69)	0.165
Solid tumor	59 (3.81)	18 (2.49)	0.104
Chronic renal failure	56 (3.62)	19 (2.62)	0.218
Liver cirrhosis	48 (3.10)	10 (1.38)	0.016
Autoimmune disease receiving treatment	86 (5.55)	31 (4.28)	0.202
Transplantation receiving treatment	25 (1.61)	4 (0.55)	0.036
Diabetes mellitus	79 (5.10)	37 (5.11)	0.992
Confirmed ATB	1091 (70.43)		
Xpert	893 (57.65)		
Culture	836 (53.97)		
Probable ATB	458 (29.57)		

Data are presented as numbers (%) unless otherwise indicated

*TB* tuberculosis, *ATB* active tuberculosis, *SD* standard deviation, *AFS* acid-fast staining, *PCR* polymerase chain reaction

No significant difference was found in sex, human immunodeficiency virus infection, hematologic malignancy, solid tumor, chronic renal failure, autoimmune disease receiving treatment, diabetes mellitus. The rate of history of TB in the ATB group was higher than that in the non-TB group. More liver cirrhosis in ATB group than non-TB group (P = 0.016), and transplantation receiving treatment (P = 0.036) (Table 1).

### Diagnostic performance of the T-SPOT.TB assay and TBAg/PHA ratio for active TB

**Fig. 2:**
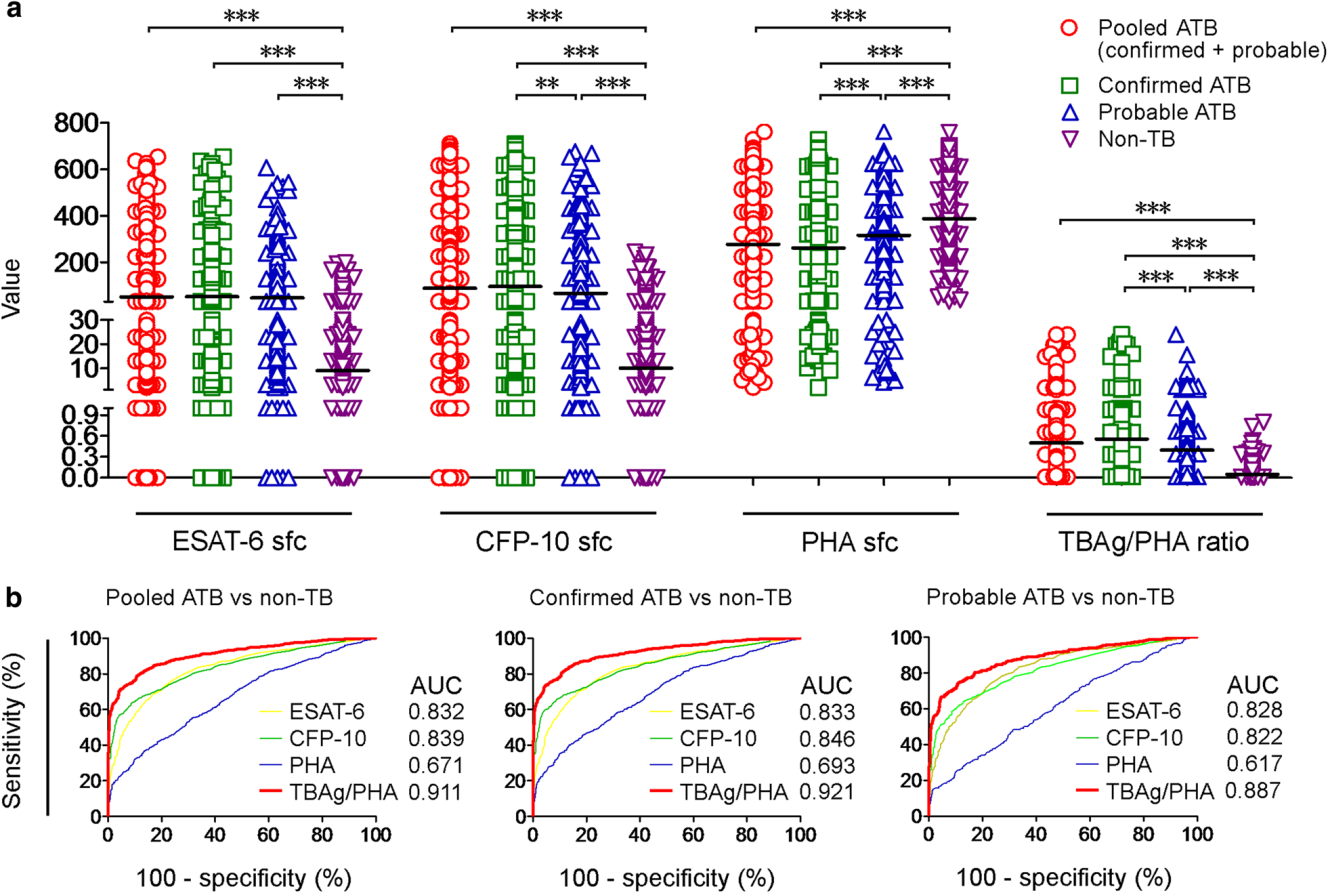
ESAT-6, CFP-10 and PHA IFN-γ response results

We observed a significant increase in the results of ESAT-6in ATB group compared with non-TB (P < 0.0001) (Fig. 2A, Table 2). The mean value of ESAT-6 were 90 ± 107.2 (mean ± SD) sfc in ATB group, and 16.9 ± 23.91 sfc in non-TB group. The mean value of CFP-10 were 150.1 ± 161.7 sfc in ATB group and 18.48 ± 27.59 sfc in non-TB group. There was also a significant increase in the results of CFP-10 in ATB group compared with non-TB (P < 0.0001) (Fig. 2A, Table 2).

**Table 2:** ROC analysis of different indicators in distinguishing ATB from non-TB in suspected patients with positive T-SPOT results

	Cutoff value	Sensitivity	Specificity	PLR	P value
ESAT-6 sfc	> 20	76.4	76.0	3.2	P < 0.0001
CFP-10 sfc	> 40	66.4	88.7	5.9	P < 0.0001
PHA sfc	< 214	37.3	85.8	2.6	P < 0.0001

ATB including confirmed ATB and probable ATB

*ROC* receiver operating characteristic, *ATB* active tuberculosis, *ESAT-6* early secreted antigenic target 6, *CFP-10* culture filtrate protein 10, *PHA* phytohaemagglutinin, *sfc* spot-forming cells

Compared the results of ESAT-6 in Confirmed ATB group (mean ± SD: 92.67 ± 109.6 sfc) with non-TB group, and Probable ATB group (83.63 ± 101 sfc) with non-TB group, there were statistically significant differences (both P < 0. 001). Compared the results of CFP-10 in Confirmed ATB group (mean ± SD: 160 ± 166.8 sfc) with non-TB group, and Probable ATB group (126.4 ± 146.1 sfc) with non-TB group, there were statistically significant differences (both P < 0. 001).

There was a significant decrease in the results of PHA in ATB group compared with non-TB (P < 0.0001) (Fig. 2A, Table 2). The mean value of PHA was 286.3 ± 165.5 (mean ± SD) SFCs/2.5 × 10^5^ in ATB group, and 387.1 ± 152.2 SFCs/2.5 × 10^5^ in non-TB group. Compared the results of PHA in confirmed ATB group (273.9 ± 163.3 SFCs/2.5 × 10^5^) with non-TB group, and probable ATB group (315.9 ± 167 SFCs/2.5 × 10^5^) with non-TB group, there were statistically significant differences (both P < 0. 001).

The AUC of the receiver operating characteristic (ROC) curve for ESAT-6 was 0.832 (95% CI 0.814–0.849) in distinguishing ATB from non-TB, CFP-10 was 0.839 (95% CI 0.823–0.855), and PHA was 0.671 (95% CI 0.648–0.694) (Fig. 2B, Table 2). Meanwhile, we compared the confirmed ATB and Probable ATB *vs* non-TB, the results were similar (Fig. 2A, Table 2).

We calculated the TBAg/PHA ratio of every individual (the larger value of ESAT-6 sfc to PHA sfc or CFP-10 sfc to PHA sfc). We observed a significant increase in the results of TBAg/PHA ratio (P < 0.0001) in ATB group compared with non-TB group (Fig. 2B). The median values and IQR of TBAg/PHA ratio were 0.5 (0.192–1.10) and 0.052 (0.029–0.097) in the ATB group and the non-TB group. The AUC of ROC curve for TBAg/PHA ratio was 0.911 (95% CI 0.899–0.922) in distinguishing ATB from non-TB (Fig. 2B).

These data suggest that directly using PHA results has no enough value (AUC = 0.671) in distinguishing the ATB and the non-TB. The AUC of ESAT-6 and CFP-10 were performed a certain value in distinguishing the two group, but the TBag/PHA ratio showed outstanding performance. Therefore, we focus on a comprehensive analysis of the TBag/PHA ratio.

Table 2 ROC analysis of different indicators in distinguishing ATB from non-TB in suspected patients with positive T-SPOT results.

### The performance of different thresholds of Tbag/PHA ratio for ATB diagnosis

According to the statistical analysis of ROC curve, the TBag/PHA ratio in distinguishing the ATB group and the non-TB group showed that the best threshold value was 0.1443. When the threshold value of 0.1443 was used, the sensitivity was 81.73% (95% CI 79.71–83.63%%)and the specificity was 86.6% (95% CI 83.9–89%), giving a PPV of 92.9 (95% CI 91.5–94.0%) and a PLR of 6.1 (95% CI 5.1–7.3) for ATB. This optimal threshold (0.1443) may show the best balance between sensitivity and specificity, but we generally need better specificity and sometimes need better sensitivity. Therefore, we calculated the performance of different thresholds, as shown in Table 3.

**Table 3:** The performance of different thresholds of Tbag/PHA ratio for ATB diagnosis

TBag/PHA threshold	Sensitivity (95%CI)	Specificity (95%CI)	PPV (95%CI)	NPV (95%CI)	PLR (95%CI)	NLR (95%CI)
0.1443	81.7 (79.6–83.6)	86.6 (83.9–89.0)	92.9 (90.9–94.9)	68.8 (66.8–70.8)	6.1 (5.1–7.3)	0.2 (0.2–0.2)
0.1817	76.2 (74.0–78.3)	90.1 (87.6–92.1)	94.3 (92.3–96.3)	63.9 (61.9–65.9)	7.7 (6.1–9.6)	0.3 (0.2–0.3)
0.2004	74.4 (72.1–76.5)	92.3 (90.1–94.1)	95.4 (93.4–97.4)	62.7 (60.7–64.7)	9.6 (7.5–12.4)	0.3 (0.3–0.3)
0.0783	90.1 (88.5–91.5)	67.8 (64.3–71.2)	85.7 (83.7–87.7)	76.1 (74.1–78.1)	2.8 (2.5–3.1)	0.1 (0.1–0.2)

According to ROC analysis, if a specificity of more than 90% will be obtained, TBag/PHA ratio threshold value of 0.1817 should be used to discriminate between ATB and non-TB. When TBag/PHA ratio threshold value was 0.2004, the specificity was 92.27% (95% CI 90.07–94.1%) and the sensitivity was 74.37% (95% CI 72.12–76.53%) (Table 3).If sensitivity of more than 90% will be obtained, a threshold value of 0.0783 should be used, the sensitivity was 90.06% (95% CI 88.46–91.5%) and the specificity was 67.82% (95% CI 64.28–71.21%). Compared TBag/PHA ratio in the confirmed ATB group or probable ATB group vs non-TB group, the ROC analysis results were similar (Fig. 2B, Table 3).

### The TBAg/PHA ratio and diseases affecting host immune status

**Fig. 3:**
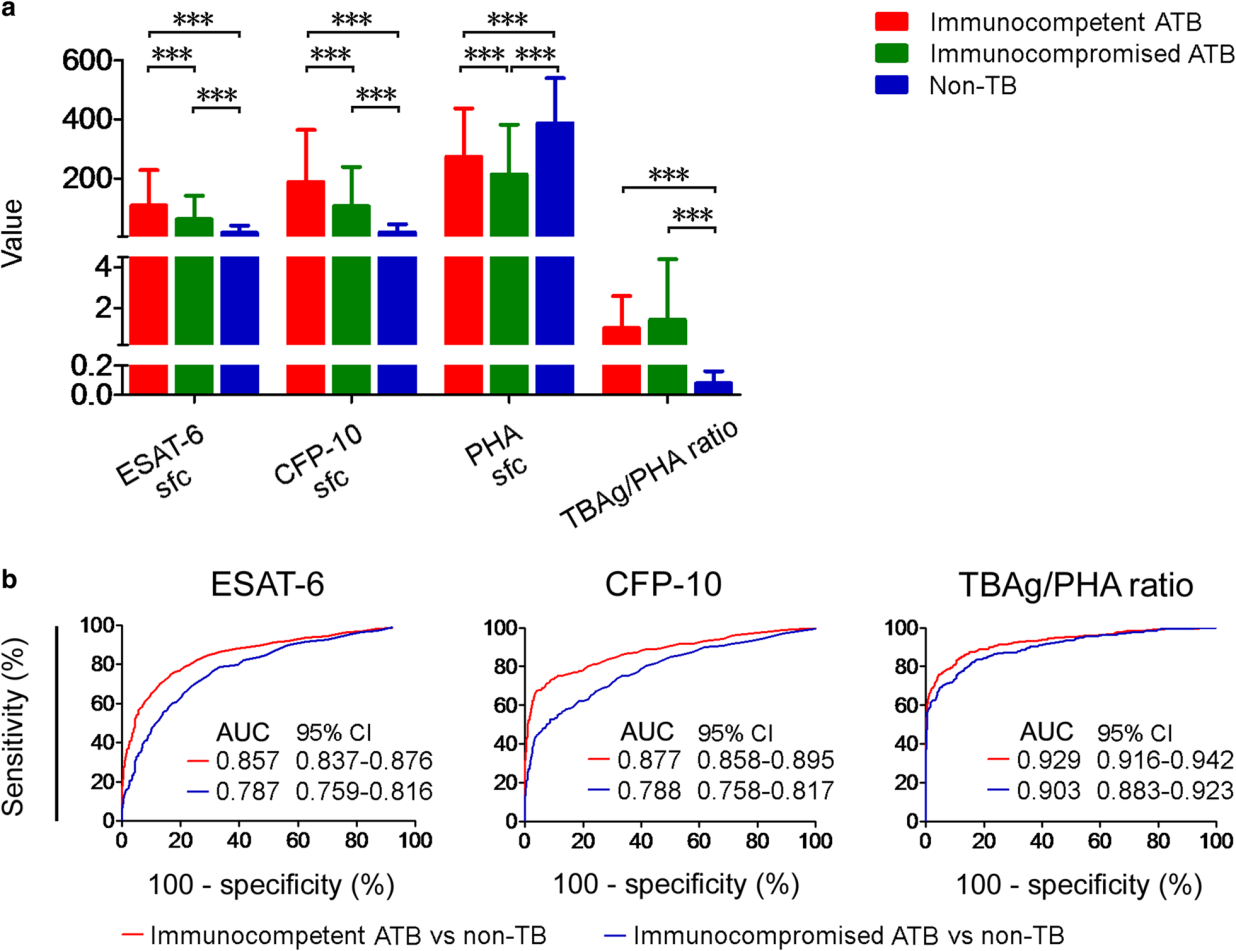
ESAT-6, CFP-10, PHA and TBAg/PHA ratio results in immunocompromised and immunocompetent ATB patients

We analyzed the relationship between the results of T-SPOT.TB assay and diseases affecting host immune status. The underlying conditions listed in Table 1 were considered as immunosuppressive status, such as HIV infection, autoimmune disease receiving treatment, etc. The results of ESAT-6 and CFP-10 in immunocompromised patients were lower than others, while ESAT-6 and CFP-10 results in non-TB patients were even lower (Fig. 3A). But the results of PHA were different, PHA value increased in non-TB patients (Fig. 3A). The result of TBAg/PHA ratio showed that it was the highest among immunocompromised patients. ROC analysis showed that compared with ESAT-6 and CFP-10, the AUC of Tbag/PHA ratio in immunocompromised ATB and non-TB was closer, suggesting that Tbag/PHA ratio is less affected by the immune status of the host (Fig. 3B).

### The TBAg/PHA ratio and the types of ATB and patient's condition

**Fig. 4:**
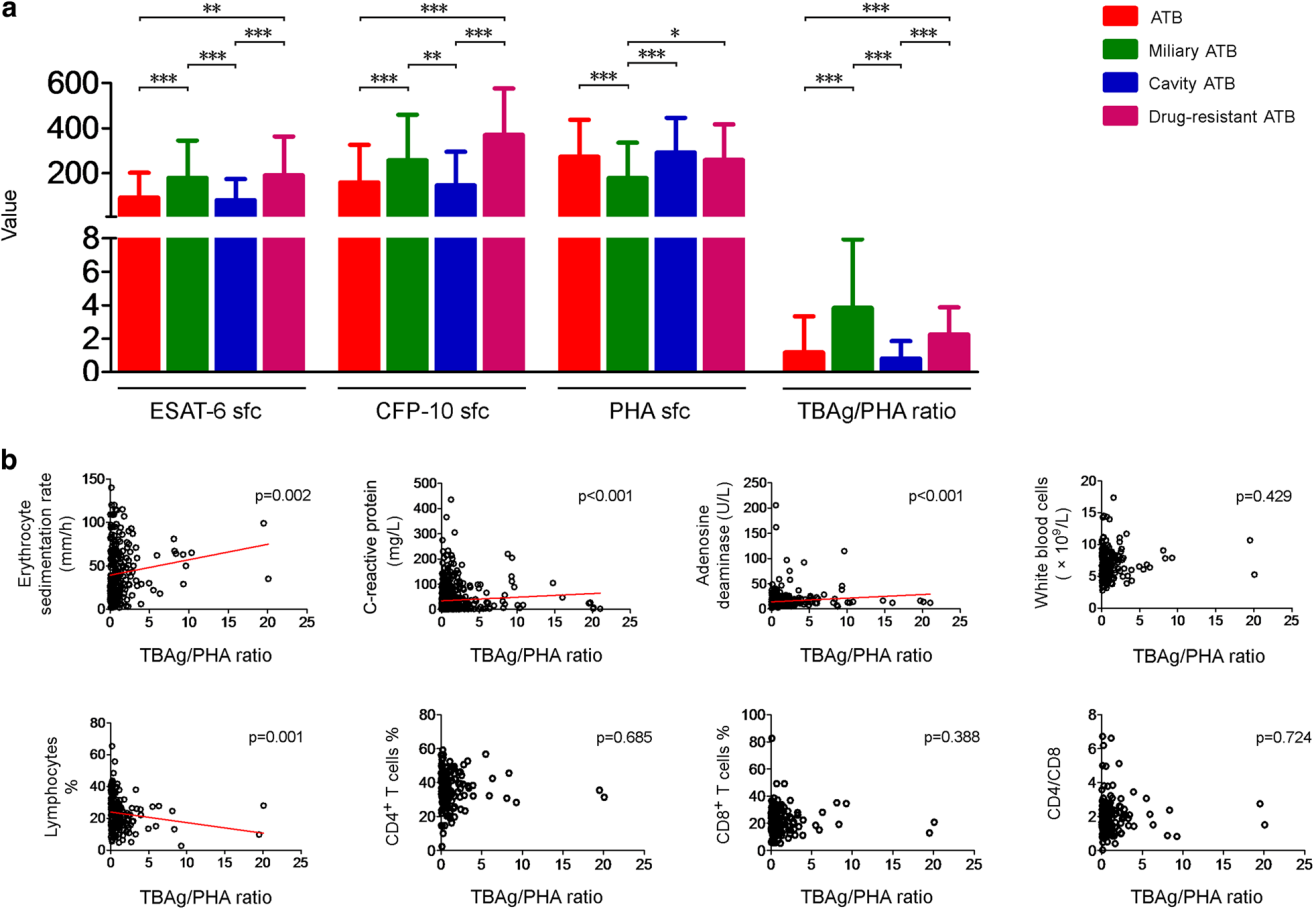
ESAT-6, CFP-10, PHA and TBAg/PHA ratio results and the activity of disease

Some types of tuberculosis or the patient's condition shows that their immune status was different from that of general ATB patients, T-SPOT assay's results can reflect it. We observed that ESAT-6 sfc, CFP-10 sfc, and TBAg/PHA ratio were increased in miliary tuberculosis patients and drug-resistant ATB patients compared with all ATB patients (Fig. 4A). But it was not observed in patients with cavitary pulmonary tuberculosis (cavity ATB) (Fig. 4A). Furthermore, we observed that the TBAg/PHA ratio was positively correlated with erythrocyte sedimentation rate(ESR), C-reactive protein, and peripheral blood adenosine deaminase in ATB patients, while it was negatively correlated with the percentage of lymphocytes (Fig. 4B). However, we did not observe a significant correlation between TBAg results with these indicators (Fig. 4).

## Discussion

The positive rate of pathogenic examination about tuberculosis is low, which makes it difficult for many tuberculosis patients to early diagnose. Meanwhile, there are few other early identification methods. Interferon-gamma release assays (IGRA) for specific immunological diagnosis of Mycobacterium tuberculosis infection have become available recently. But the value of IGRA in the diagnosis of active tuberculosis remains controversial. Several studies have demonstrated that IGRAs may be useful as supplemental tools in the diagnosis of active TB [23, 24].T-SPOT.TB assay is one of the two commercial IGRA assays, recent studies have reported that T-SPOT.TB has a certain diagnostic value for active pulmonary tuberculosis by increasing the threshold [25].

The results of this study showed that ESAT-6, CFP-10 and PHA in the T-SPOT.TB assay are helpful to distinguish ATB and non-TB ((both P < 0.0001) (Table 2). But the overall specificity of three indicators is moderate, the sensitivity is slightly poor.

In our previous studies, we have proposed a new method based on the calculation of the ratio of TBAg to PHA in the T-SPOT analysis [20, 21]. Through the retrospective study [21] (162 active TB patients and 97 LTBI), we concluded that the TBAg/PHA ratio (the higher value of ESAT-6/PHA or CFP-10/PHA ratios) in active TB patients were significantly higher than in individuals with LTBI. Therefore, we consider that the value of Tbag/PHA ratio may be helpful in the diagnosis of active tuberculosis. Therefore, the diagnostic utility of TBAg/PHA ratio was validated through this multicenter prospective study.

ROC analysis shows that ESAT-6, CFP-10, and PHA all have good AUC (all above 85%), but the AUC of the TBAg/PHA ratio can reach more than 90%. ROC analysis showed that using the best threshold 0.1413, the sensitivity was 81.73% (95% CI 79.71–83.63%%) and the specificity was 86.6% (95% CI 83.9–89%). When using the threshold value of 0.1817, the specificity was 90.06% (95% CI 87.64–92.14%), with a good PPV and a PLR for ATB, which can reached a good clinical application value. When the TBag/PHA ratio threshold value 0.2004 was used, the specificity can be further increased, the specificity was 92.3% (95% CI 90.1–94.1%). Threshold value 0.200 is a relatively simple and accurate value, which is convenient for calculation and used with very good specificity and acceptable sensitivity (Fig. 2B).

As a detection of the body's immune response, the results of the T-SPOT.TB assay will be affected by the body's immune state (Table 1). But the TBAg/PHA ratio were not affected by the underlying conditions. Our previously study reported that even in the immunocompromised patients, the TBAg/PHA ratio have some significance for the diagnosis of TB disease [22], the results of this study are consistent with it.

However, our study also has some shortcomings. There were 1339 (35.9%) patients with negative T-SPOT results were excluded from this study. Some proportion of them may eventually be diagnosed with ATB. It has been reported that IGRA may be affected by NTM. In this study, 86 cases of NTM were identified through mycobacterium culture, of which 21 cases (24.4%) were T-SPOT positive. No further analysis was made due to the small number of cases.

## Conclusions

This prospective, multi-center clinical study reveals that the TBAg/PHA ratio in T-SPOT.TB Assay shows high prospective value in the diagnosis of Active Tuberculosis in T-SPOT positive patients. Especially when the TBag/PHA ratio threshold is 0.2004, good specificity and PLR can be obtained. The TBAg/PHA ratio are less affected by diseases affecting host immune status. By calculating the TBAg/PHA ratio, this method might break through the limitation of using T-SPOT assay in in high-TB prediction areas and may help early diagnosis of ATB.

## Data Availability

Not applicable.

## Abbreviations

IGRAs: Interferon-gamma release assays
Mtb: Mycobacterium tuberculosis
ATB: Active tuberculosis
PHA: Phytohaemagglutinin
ESAT-6: Early secreting antigenic target-6
CFP-10: Culture filtrate protein-10
LTBI: Latent tuberculosis infection
WHO: World Health Organization
NTM: Non-tuberculous mycobacteria
